# Immune Thrombocytopenic Purpura: Late-Onset Thrombocytopenia Following Mild COVID-19 Infection

**DOI:** 10.7759/cureus.113527

**Published:** 2026-07-28

**Authors:** Hakan Karatas, Ozlem Ozdemir Candan, Banu Ocak, Tayfur Toptas, Isik Kaygusuz Atagündüz

**Affiliations:** 1 Allergy and Immunology, İzmir Şehir Hastanesi, İzmir, TUR; 2 Hematology, Marmara University Pendik Training and Research Hospital, Istanbul, TUR; 3 Internal Medicine, Marmara University Pendik Training and Research Hospital, Istanbul, TUR

**Keywords:** covid-19, immune mediated thrombocytopenic purpura (itp), intravenous immunoglobulin, sars-cov-2, thrombocytopenia

## Abstract

Immune thrombocytopenic purpura (ITP) is an acquired autoimmune disorder characterized by immune-mediated platelet destruction and impaired platelet production. Secondary ITP may occur following infections, including coronavirus disease 2019 (COVID-19); while thrombocytopenia is frequently observed in severe COVID-19, delayed-onset ITP after a mild, non-hospitalized infection is uncommon. We report a previously healthy 35-year-old man who presented with spontaneous epistaxis, widespread petechiae, and oral hemorrhagic bullae 14 days after a mild, home-managed COVID-19 infection. Laboratory evaluation revealed isolated severe thrombocytopenia (platelet count 2 x 10³/μL) with normal hemoglobin, leukocyte count, coagulation parameters, and peripheral blood smear; autoimmune and infectious workup was negative, and a prior platelet count had been normal. COVID-19-associated secondary ITP was diagnosed. Given high-risk bleeding manifestations, oral prednisone (1 mg/kg/day) and a single dose of intravenous immunoglobulin (1 g/kg) were initiated, with platelet counts increasing to 43 x 10³/μL by day 5 and normalizing within one week, remaining stable at day 75. This case highlights that severe ITP may develop during the recovery phase of even mild COVID-19, underscoring the importance of clinical vigilance and prompt immunosuppressive therapy.

## Introduction

Immune thrombocytopenic purpura (ITP) is an acquired hematologic disorder characterized by immune-mediated destruction of platelets and impaired platelet production from megakaryocytes. The estimated incidence in adults is approximately two to four cases per 100,000 persons per year, and the clinical spectrum ranges from asymptomatic thrombocytopenia discovered incidentally to mucocutaneous bleeding and, less commonly, life-threatening hemorrhage [1,2]. When associated with an underlying condition such as infection or autoimmune disease, ITP is classified as secondary [1,2]. An association between COVID-19 infection and secondary ITP has been recognized, but reports describing severe thrombocytopenia following mild, non-hospitalized COVID-19 infection remain limited [3]. Herein, we present a case of late-onset severe immune thrombocytopenic purpura developing after a mild COVID-19 infection, and we describe the diagnostic reasoning and therapeutic approach.

## Case presentation

A 35-year-old Caucasian male presented to the emergency department with a four-day history of spontaneous epistaxis unresponsive to nasal packing, widespread petechiae on the extremities, and hemorrhagic bullae on the lower lip. The patient, a physics teacher working remotely for the past year, had no known chronic illnesses, except for an incidental finding of gallstones on an abdominal radiograph five years prior. In January 2021, 14 days before presentation, he developed sudden-onset fatigue, chills, and upper respiratory tract symptoms, and his COVID-19 polymerase chain reaction test was positive. Because his symptoms were mild, he was isolated at home for 10 days. He received favipiravir (3,200 mg on day one, followed by 1,200 mg/day divided into two doses for the next four days), which was the treatment recommended in Türkiye at that time. He did not receive azithromycin or hydroxychloroquine. During isolation, his body temperature remained below 38.5°C, and his peripheral oxygen saturation, measured with a personal pulse oximeter, remained above 95%. He had not received any COVID-19 vaccine before or during this period; national vaccination had begun only days earlier and was restricted to healthcare workers, so vaccine-associated immune thrombocytopenia could be excluded on temporal grounds. The patient self-reported taking a daily multivitamin supplement and 100 mg/day of acetylsalicylic acid (ASA). He denied the use of other over-the-counter medications, herbal products, or illicit substances.

At the emergency department, his vital signs were stable (blood pressure 107/78 mmHg, pulse 81 bpm, temperature 36.5 C). Physical examination revealed two hemorrhagic bullae on the lower lip and two additional lesions near the lower left first premolar and first molar. Petechial rash was widespread, especially on the lower extremities, and venipuncture sites were surrounded by ecchymosis. No hepatosplenomegaly or lymphadenopathy was detected. Laboratory evaluation revealed isolated severe thrombocytopenia (platelet count 2 x 10³/μL) with normal hematological indices and coagulation parameters; the complete laboratory findings, reference ranges, and units are presented in Table 1. A peripheral blood smear showed decreased platelets without clumping, schistocytes, or blast cells.

**Table 1:** Laboratory findings at presentation with corresponding reference ranges and units

Parameter	Patient value	Reference range	Units
White blood cell count	7.4	4.0-10.0	x10³/μL
Neutrophils	5.0	2.0-7.0	x10³/μL
Lymphocytes	1.6	1.0-3.0	x10³/μL
Hemoglobin	14.9	13.5-17.5	g/dL
Hematocrit	41.9	40-52	%
Mean corpuscular volume	86.7	80-100	fL
Platelet count	2	150-450	x10³/μL
Prothrombin time	13.4	11-13.5	s
Partial thromboplastin time	28.8	25-35	s
D-dimer	0.39	0-0.5	mg/L
Fibrinogen	491	200-400	mg/dL
Lactate dehydrogenase	523	0-248	U/L

Autoimmune and infectious screening revealed negative results for antinuclear antibodies, rheumatoid factor, anti-cyclic citrullinated peptide, anti-Sjögren's syndrome-related antigen A (SSA), anti-Sjögren's syndrome-related antigen B (SSB), anti-topoisomerase I antibody (Scl70), anti-double-stranded DNA (dsDNA), anti-β2 glycoprotein, and anticardiolipin antibodies. Serologies for hepatitis B virus, hepatitis C virus, human immunodeficiency virus, cytomegalovirus, Epstein-Barr virus, varicella-zoster virus, and parvovirus B19 were negative. Both direct and indirect Coombs tests were negative.

The patient reported no prior history of bleeding diathesis, petechiae, purpura, or bruising. A complete blood count one year prior had documented a normal platelet count of 169 x 10³/μL, ruling out congenital thrombocytopenia or a chronic hematologic condition. Given the clinical presentation and laboratory findings, a diagnosis of secondary immune thrombocytopenic purpura associated with COVID-19 was established. Acetylsalicylic acid was discontinued at admission. The presence of oral hemorrhagic bullae, considered a marker of increased risk of life-threatening hemorrhage [4], prompted urgent treatment with oral prednisone (1 mg/kg/day) and a single dose of intravenous immunoglobulin (IVIg; 1 g/kg).

**Figure 1:**
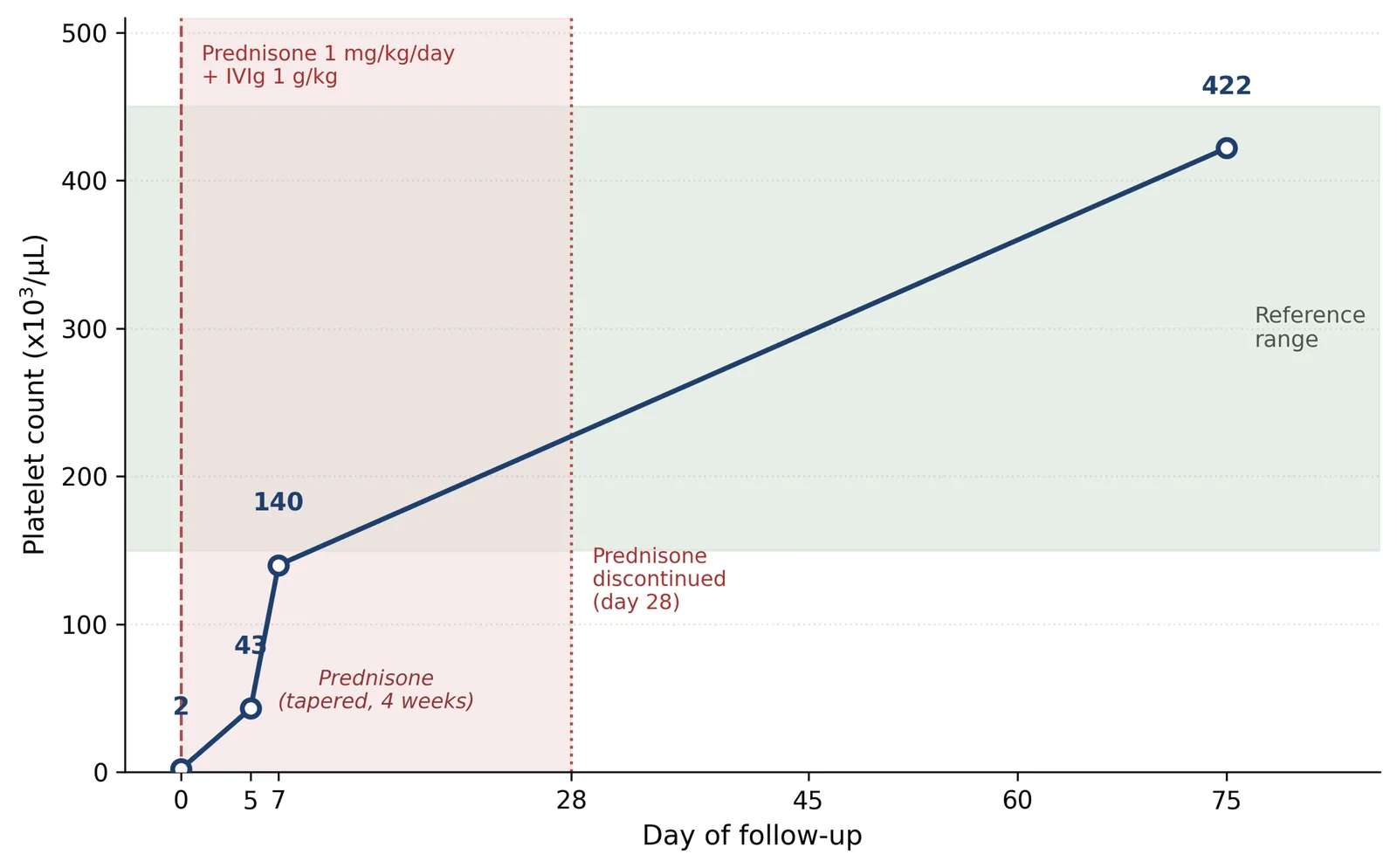
Platelet count trajectory from admission to day 75 The green band indicates the laboratory reference range (150-450 x 10³/μL), and the shaded red area indicates the period of prednisone therapy, which was tapered and discontinued by the end of the fourth week. The dashed line marks initiation of oral prednisone (1 mg/kg/day) and a single dose of intravenous immunoglobulin (1 g/kg) on the day of admission. The day 75 platelet count of 422 x 10³/μL was recorded off all therapy.

By the fifth day of treatment, the petechial rash had resolved and the platelet count increased to 43 x 10³/μL. The platelet count reached 140 x 10³/μL at one week, marginally below the lower reference limit. Once a hemostatically safe platelet count had been achieved, prednisone was tapered stepwise and discontinued by the end of the fourth week, consistent with guideline recommendations to limit corticosteroid exposure in newly diagnosed ITP to a short course of six weeks or less including taper [5]. The patient was therefore receiving no ITP-directed therapy at the day 75 assessment, at which the platelet count was 422 x 10³/μL with no recurrence of bleeding symptoms, indicating a sustained response off treatment (Figure 1). The patient tolerated all treatments well, with no adverse events, and no changes to the therapeutic regimen were required. Written informed consent was obtained from the patient for publication of this case report and any accompanying data. This case report was prepared in accordance with the Case Report (CARE) reporting guidelines.

## Discussion

In patients with COVID-19, especially those hospitalized and receiving prophylactic or therapeutic heparin to reduce thrombotic risk, new-onset thrombocytopenia should prompt evaluation for heparin-induced thrombocytopenia [6]. However, the patient had no history of heparin exposure. Although favipiravir has been associated with neutropenia, it has not been linked to thrombocytopenia [7]. Low-dose acetylsalicylic acid impairs platelet function but does not reduce the platelet count. Vaccine-associated immune thrombocytopenia, a recognized entity, was excluded because the patient had not received any COVID-19 vaccine. The comprehensive exclusion of alternative etiologies, together with a documented normal pre-illness platelet count, supports COVID-19 as the trigger in this case.

The serum lactate dehydrogenase level of 523 U/L was more than twice the upper reference limit. In the absence of anemia, this elevation is most consistent with a residual acute-phase response during convalescence from COVID-19. Hemolysis as an alternative explanation was excluded based on the normal hemoglobin concentration, negative direct and indirect Coombs tests, and the absence of schistocytes on the peripheral smear; these findings also argued against thrombotic microangiopathy.

An important management consideration is the contribution of acetylsalicylic acid to the hemorrhagic phenotype. Although ASA does not cause thrombocytopenia, its irreversible inhibition of platelet cyclooxygenase-1 abolishes thromboxane-mediated aggregation in the small number of circulating platelets that remain. In the setting of a platelet count of 2 x 10³/μL, this pharmacological platelet dysfunction plausibly potentiated the severity of the bleeding manifestations, including the refractory epistaxis and wet purpura. ASA was therefore discontinued at admission. Because the antiplatelet effect of aspirin persists for the lifespan of the exposed platelets, clinicians should specifically elicit a history of over-the-counter antiplatelet use in any patient presenting with severe thrombocytopenia and disproportionate bleeding.

Thrombocytopenia in COVID-19 can result from multiple mechanisms, including disseminated intravascular coagulation, endothelial damage, molecular mimicry, cytokine storm-induced immune dysregulation, and impaired megakaryopoiesis [8]. SARS-CoV-2 enters host cells principally through the angiotensin-converting enzyme 2 receptor with transmembrane serine protease 2 priming. Autopsy and in vitro studies have demonstrated infection of lung megakaryocytes and platelets, together with megakaryocyte-rich microthrombi in pulmonary, cardiac, and cerebral capillaries, providing a plausible link between viral infection and disordered thrombopoiesis [9].

COVID-19-associated ITP has been reported in a growing number of series, with thrombocytopenia typically developing 13 to 14 days after the onset of COVID-19 symptoms [10,11]. In a systematic review and meta-analysis of COVID-19-associated immune thrombocytopenia, the majority of affected patients had comorbidities or severe COVID-19, and only a minority developed ITP following a mild course of infection [11,12]. This case is notable because the patient developed severe thrombocytopenia during the recovery phase after a mild COVID-19 infection, without hospitalization and without any comorbidity, indicating that acute disease severity does not reliably predict hematological risk.

In this patient, the presence of oral hemorrhagic bullae ("wet purpura") indicated a high risk for life-threatening bleeding and prompted urgent immunosuppressive therapy. Treatment with corticosteroids and intravenous immunoglobulin resulted in rapid hematologic recovery, and the response proved durable: the platelet count of 422 x 10³/μL at day 75 was recorded after prednisone had been discontinued for approximately seven weeks, distinguishing a genuine sustained remission from a count maintained on ongoing corticosteroid therapy. This is clinically relevant because corticosteroid dependence would have implied a different disease trajectory and the need for second-line therapy. A key strength of this report is the comprehensive exclusion process and the availability of a normal pre-illness platelet count. A limitation is the absence of anti-platelet antibody testing.

## Conclusions

Severe immune thrombocytopenic purpura can develop after mild COVID-19 infection, often presenting during the recovery phase in previously healthy individuals. Clinicians should maintain a high index of suspicion for new-onset bleeding manifestations in post-COVID patients, regardless of initial disease severity, and should specifically ascertain concurrent antiplatelet use, which may amplify the hemorrhagic phenotype. Early diagnosis and timely initiation of immunosuppressive therapy, such as corticosteroids and intravenous immunoglobulin, are crucial to prevent life-threatening hemorrhagic complications.

## References

[1] Thakre R, Gharde P, Raghuwanshi M (2023). Idiopathic thrombocytopenic purpura: current limitations and management. Cureus.

[2] Madkhali MA (2024). Recent advances in the management of immune thrombocytopenic purpura (ITP): a comprehensive review. Medicine.

[3] Alonso-Beato R, Morales-Ortega A, Fernández FJ, Morón AI, Ríos-Fernández R, Rubio JL, Centeno NO (2021). Immune thrombocytopenia and COVID-19: case report and review of literature. Lupus.

[4] Bezerra Machado AI, Vieira Fernandes I, Araújo M, Pinto L (2024). 'Wet purpura': a sign of immune thrombocytopenia. Intern Emerg Med.

[5] Neunert C, Terrell DR, Arnold DM (2019). American Society of Hematology 2019 guidelines for immune thrombocytopenia. Blood Adv.

[6] Abudouleh E, Alhamshari A, Al-Qahtani AA, Aguilos A, Owaidah T (2022). Comparison of HIT tests in patients with COVID-19 and thrombocytopenia. J Blood Med.

[7] Hashemian SM, Farhadi T, Velayati AA (2021). A review on favipiravir: the properties, function, and usefulness to treat COVID-19. Expert Rev Anti Infect Ther.

[8] Mocan M, Chiorescu RM, Tirnovan A, Buksa BS, Farcaș AD (2022). Severe thrombocytopenia as a manifestation of COVID-19 infection. J Clin Med.

[9] Zhu A, Real F, Capron C (2022). Infection of lung megakaryocytes and platelets by SARS-CoV-2 anticipate fatal COVID-19. Cell Mol Life Sci.

[10] Fu H, Cai X, Cui L (2024). The evolution of preexisting primary immune thrombocytopenia after COVID-19 onset: a nationally representative, prospective, multicentre, observational study. Ann Hematol.

[11] Alharbi MG, Alanazi N, Yousef A, Alanazi N, Alotaibi B, Aljurf M, El Fakih R (2022). COVID-19 associated with immune thrombocytopenia: a systematic review and meta-analysis. Expert Rev Hematol.

[12] Taherifard E, Taherifard E, Movahed H, Mousavi MR (2021). Hematologic autoimmune disorders in the course of COVID-19: a systematic review of reported cases. Hematology.

